# The Relationship between 24 h Ultramarathon Performance and the “Big Three” Strategies of Training, Nutrition, and Pacing

**DOI:** 10.3390/sports10100162

**Published:** 2022-10-21

**Authors:** Fuminori Takayama, Hisashi Mori

**Affiliations:** School of Human Science and Environment, University of Hyogo, Himeji 670-0092, Japan

**Keywords:** extreme endurance, continuous glucose monitoring, sports nutrition, heart rate

## Abstract

Background: The present case study examined the relationship between 24 h ultramarathon performance and the “big three” strategies of training, nutrition, and pacing. Methods: A 32-year-old male ultramarathon runner (body mass: 68.5 kg, height: 179 cm) participated in a 24 h ultramarathon race. Training status was quantified based on from a GPS sports watch. The nutritional status was evaluated during the week leading up to the race, and blood glucose level and heart rate were measured during the race. Results: His aim of the distance was 200 km, but the actual performance was 171.760 km. The blood glucose level was stable because of adequate CHO intake before (7.2 ± 0.8 g/kg/day) and during the race (48 g/h). The running speed decreased in the middle and later stages of the race despite adequate CHO intake and a lack of high intensity running in the early stage of the race. The longest training session before the race (80 km) had to be significantly shorter compared to the aim. Conclusions: For optimal 24 h ultramarathon performance, the “big three” strategies of training, nutrition, and pacing are all important. However, the performance level estimated based on previous studies may be achievable even with insufficient training, as long as the nutritional and pacing strategies are appropriate.

## 1. Introduction

An ultramarathon is defined as a race with a distance greater than a full marathon (42.195 km) and includes varieties based on distance (e.g., 50 km and 100 km) or time (e.g., 12 h and 24 h) limit. World championships for the popular and highly competitive 24 h ultramarathons are organized by the International Association of Ultrarunners.

The “big three” strategies of training, nutrition, and pacing are known to influence performance in 24 h ultramarathons. Knechtle et al. [1] reported that 24 h ultramarathon performance is significantly related to personal best marathon time and longest training session before the race, suggesting the need for adequate training. High aerobic fitness runners have the advantage of lower relative intensities compared to low aerobic fitness runners while running at the same absolute intensities (running speed). Since ultramarathon running causes severe muscle damage [2], adequate training to prevent muscle damage is required for success.

Carbohydrate (CHO) intake is an important nutritional factor for ultramarathon success. A previous study of food and fluid intake during the World Championship reported that performance positively correlated with energy intake [3]. Successful ultramarathon performance is generally associated with greater CHO consumption [3,4,5,6]. This is explained by the large CHO quantities consumed during the race and the limited amount of glycogen in the liver and muscles. Although international-level ultramarathon runners could consume over 60/h of CHO [3], such a high intake may be difficult for recreational ultramarathon runners due to nutrient malabsorption and gastrointestinal distress [7]; therefore, 30–50 g/h of CHO intake is recommended for single ultramarathons [8].

Several studies have recently investigated blood glucose levels during ultramarathons using the flash glucose monitoring system [4,5,6]. Ishihara et al. [6] evaluated blood glucose levels and nutrition strategies in seven runners during a 160 km ultramarathon and reported that runners with low CHO intake tended to have lower blood glucose levels. Another field study investigated the relationship between CHO intake, blood glucose levels, and performance during a 24 h ultratrail race and reported that pre- and in-race CHO intake was significantly related to performance [4]. However, it also reported that the mean glucose level did not correlate with performance or CHO intake [4]. These conflicting results suggest the need for additional studies to establish the relationship between CHO intake, blood glucose level, and ultramarathon performance.

The performance factors for 24 h ultramarathons are interdependent. Since exercise intensity affects internal organ blood flow, over-pacing may cause gastrointestinal distress and subsequently low CHO intake. Even with sufficient CHO consumption, an insufficiently trained runner may not be able to maintain speed during the second half of the race. Although an increasing number of studies have explored ultramarathon performance, few studies have investigated the relationships between training status, nutritional status, and pacing strategies at an individual level. The present case study examined the relationship between 24 h ultramarathon performance and the “big three” strategies of training, nutrition, and pacing.

## 2. Materials and Methods

### 2.1. Participant

A 32-year-old male ultramarathon runner (body mass: 68.5 kg, height: 179 cm) participated in the present case study. He had completed more than 10 full marathons and several ultramarathons (including 6 h, 12 h, 24 h, and 100 km). However, most of the runner’s ultramarathon experience was >7 years ago; he resumed training for a 24 h ultramarathon half a year ago. His personal best records were 2:57:00 h, 8:57:21 h, 184.691 km for the full marathon (February 2020), 100 km ultramarathon (June 2014), and 24 h ultramarathon (November 2014), respectively. Informed consent was obtained, and the study was approved by the School of Human Science and Environment, University of Hyogo Research Ethics Committee (no. 277).

### 2.2. Experimental Design

The present case study was based on the 1st Hirosaki 24 h run held in Aomori, Japan. The race started at 11:00 AM on 21 May 2022, on a flat lap road and track course (1.09 km). According to the Japan Meteorological Agency, the temperature was 15.1–26.5 °C, humidity was 26–87%, and the wind speed was 0.5–3.7 m/s. The speed for each 1 h interval was calculated using the number of laps per hour. The distance for the final hour was measured manually by a race organizer. The runner aimed to reach 200 km. Since successful performance requires minimal variations throughout the 24 h period [9], the runner planned to run approximately 105 km (8.8 km/h) and 95 km (7.9 km/h) during the first and second 12 h periods, respectively.

The runner’s training status was quantified based on data from a GPS sports watch. Aerobic fitness was assessed two weeks before the race, nutritional status was evaluated during the week leading up to the race, and blood glucose level and heart rate were measured during the race.

### 2.3. Aerobic Fitness

The treadmill running test was conducted on an indoor motorized treadmill (HPT-2561S-A, Tec Gihan Co., Ltd., Uji, Japan) using a previously reported protocol [10,11] and a slope of 0%. The two-part procedure consisted of 5 min warm-up and a maximal incremental test. The velocity for the warm-up was 85% of the average velocity for the recent marathon race. After warm-up, the maximal incremental test was performed with an initial velocity of 8.4 km/h, which was increased by 0.6 km/h at 1 min intervals until volitional exhaustion.

Expired gas analysis (AE-300S; Minato Medical Science Co., Ltd., Osaka, Japan) was performed on a breath-by-breath basis using the computerized standard open circuit technique. Heart rate was measured using a Polar H7 Chest Transmitter (Polar Electro, Kempele, Finland); maximal oxygen uptake (VO2max) and maximal heart rate (HRmax) were measured as reported previously [11].

### 2.4. Nutritional Status

Nutrition intake during the week leading up to the 24 h ultramarathon was recorded using MyFitnessPal App (MyFitnessPal, Inc., San Francisco, CA, USA), which is a valid tool for analyzing short-term dietary data [12]. If dietary information was available, the runner manually entered the information into the app. Energy, protein, fat, and CHO intake was calculated in the following categories: 4–7 days before the race (baseline diet), 1–3 days before the race (pre-race diet), and on the day of the race (pre-race meal).

A nutritional plan was created for the four sections (i.e., every 6 h) before the race and the actual nutritional intake was revised within 24 h after the race. Information about the food and drinks consumed during the race was mostly obtained from the manufacturer, but some information was also obtained from another database [13].

### 2.5. Blood Glucose Level and Heart Rate during the Ultramarathon

Blood glucose concentrations were measured throughout the 24 h ultramarathon using a flash glucose monitoring system (FreeStyle Libre, Abbott Diabetes Care, Alameda, CA, USA), inserted into the upper arm subcutaneous tissue 1 h before the race. This system automatically records and stores the glucose levels every 15 min for up to 8 h. A hand-held scanning device linked to the sensor was used to synchronize the data several times during the race. Data were recorded every 15 min throughout the 24 h.

Heart rate was measured using an arm-worn optical heart rate monitor (Polar Verity Sense; Polar Electro, Kempele, Finland). The data for every second were recorded throughout the 24 h.

### 2.6. Statistical Analysis

All statistics were generated and processed using Excel for Microsoft 365 (Microsoft Corp., Redmond, WA, USA). The relationships between running speed, blood glucose level, and heart rate were analyzed using Pearson’s correlation coefficient with statistical significance at *p* < 0.05.

## 3. Results

### 3.1. Aerobic Fitness and Training Status

The VO2max and HRmax were 67.6 mL/kg/min and 187 beats/min, respectively. The weekly training distances during the five weeks before the 24 h ultramarathon were 135 km, 132 km, 100 km, 81 km, and 24 km (excluding the race), respectively. The longest training session was an 80 km long-distance session about two months before the race.

### 3.2. Race Performance

**Figure 1:**
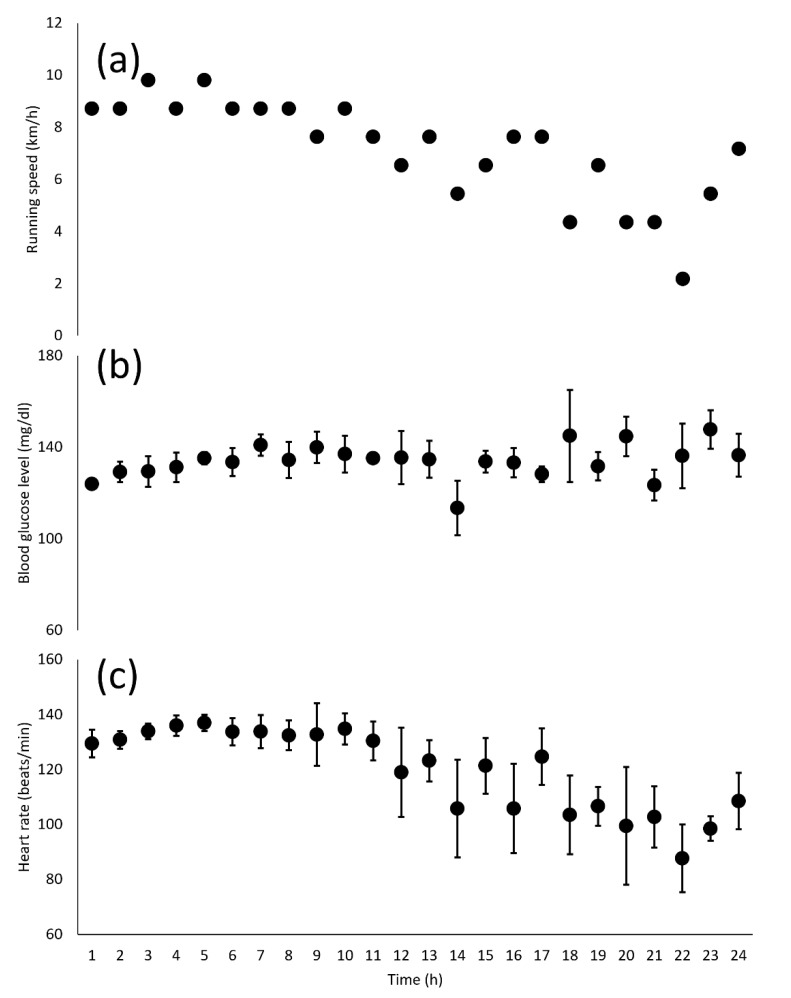
Changes in running speed (**a**), blood glucose level (**b**), and heart rate (**c**) during the 24 h ultramarathon race. Data of blood glucose level and heart rate are presented as means and standard deviations.

The total distance was 171.760 km. Figure 1a shows running speed variations during the 24 h ultramarathon. The running speed was maintained for approximately 10 h followed by a gradual decrease. The distances (speeds) for every 6 h were 54.5 km (9.1 km/h), 48.0 km (8.0 km/h), 39.2 km (6.5 km/h), and 30.1 km (5.0 km/h), respectively.

### 3.3. Nutritional Status

Table 1 shows nutritional status before the race. Compared to the baseline diet, CHO intake and percentage were increased in the pre-race diet for glycogen loading.

Table 2 shows the nutritional status during the race. The runner could eat pre-planned meals for up to half of the race. During the second half of the race, temporary mild nausea and remission of opportunity to aid stations led to slightly reduced CHO intake. However, the CHO intake remained >30 g/h. The runner consumed CHO from fluids, gels, and solids.

### 3.4. Blood Glucose Level and Heart Rate

Figure 1b,c show blood glucose level and heart rate information during the 24 h ultramarathon. The blood glucose level remained stable throughout the race, with average, minimum, and maximum values of 134 mg/dL, 114 mg/dL (14 h), and 148 mg/dL (23 h), respectively. There was no significant correlation between blood glucose level and running speed (r = −0.15).

Heart rate remained stable for up to 10 h and then decreased, with an average value of 120 ± 18 bpm (64% HRmax). The heart rates for every 6 h of the race were 134 ± 5 beats/min (71% HRmax), 131 ± 11 beats/min (70% HRmax), 117 ± 15 beats/min (62% HRmax), and 101 ± 14 beats/min (54% HRmax), respectively. There was a significant correlation between heart rate and running speed (r = 0.91, *p* < 0.05).

## 4. Discussion

The main findings of the present case study were: (a) the blood glucose level remained stable because of adequate CHO intake before and during the race; (b) despite adequate CHO intake and not high intensity running in the early stages of the race, the running speed decreased in the middle and later stages of the race; and (c) the longest training session before the race must be significantly shorter compared to the aim.

Success in ultramarathons is generally associated with greater CHO consumption [3,4,6,14]. Adequate CHO intake allows glycogen to be spared and mitigates muscle damage. It has been reported that high CHO intake (120 g/h) during a mountain marathon limited muscle damage compared to low CHO intake (60–90 g/h) [15]. However, such a high intake may be difficult in longer ultramarathons due to nutrient malabsorption and gastrointestinal distress [7]; therefore, 30–50 g/h of CHO intake is recommended for single ultramarathons [8]. Kinrade and Galloway [4] examined the dietary intake of ultramarathon runners before and during a 24 h ultratrail run and reported significantly different distances between runners with pre-race CHO consumptions ≥ 5 g/kg and < 5 g/kg (158.5 ± 30.1 km vs. 125.5 ± 21.7 km) and between those with in-race CHO consumptions ≥ 40 g/h and <40 g/h (148.4 ± 22.4 km vs. 120.2 ± 21.7 km). Another study reported that 11 international-level ultramarathon runners consumed 62.2 ± 29.6 g/h of CHO during 24 h World Championships [3]. Pre-race (7.2 ± 0.8 g/kg/day) and in-race (48 g/h) CHO consumption of the present runner were less than those for international-level ultramarathon runners [3] but were adequate according to published recommendations [8] and the Kinrade and Galloway study [4]. The runner had been undergoing long-distance training with a CHO intake of 60–90 g/h once or twice a week for about 2 months before the race. This approach is referred to as “gut training” and is often used by ultramarathon runners to maximize CHO availability and improve gastrointestinal tolerance [8]. We speculated that the runner had adequate CHO intake before and during the race, as indicated by the stable blood glucose levels.

There was no significant correlation between blood glucose level and running speed (r = −0.12), suggesting that both are not in a linear relationship. We speculate that hypoglycemia could negatively affect running speed, but hyperglycemia could not positively affect running speed for the 24 h ultramarathon due to excessive liver glycogen breakdown. This might partially explain the frequent discrepancy among previous studies about the relationship between performance and blood glucose levels.

Despite adequate CHO consumption and stable blood glucose levels, the running speed decreased 10 h into the race. The runner ran 102.5 km during the first 12 h as planned, but only ran 69.3 km during the next 12 h. We compared the heart rate with previous studies of ultraendurance sports. Neumayr et al. [16] analyzed the first race across the Alps (mean race time: 27 h 25 min) for 10 male elite cyclists and reported a %HRmax of 68% throughout the race. In addition, the %HRmax decreased from 86% during the first 6 h to 66% during the last 6 h. Another study [17] analyzed a 24 h treadmill ultramarathon for 12 male runners (mean distance: 149.2 km) and reported a %HRmax of 68%. They also reported a decrease in %HRmax from 72% during the first 6 h to 62% during the last 6 h. In the present case, the %HRmax was 64%; 71% for the first 6 h and 54% for the last 6 h, indicating a not high relative intensity during the first half of the race. Therefore, the pacing strategy of the first half was unlikely to influence running speed during the second half.

Knechtle et al. [1] investigated the determinants of 24 h ultramarathon performance from anthropometric and training perspectives in 63 male runners. They found that performance could be predicted (r^2^ = 0.46) using the following equation: performance (km) = 234.7 + 0.481 (longest training session before the race, km) − 0.594 (personal best marathon time, minutes). Interestingly, the predicted performance calculated using this equation for the present case was nearly equal to the actual performance (168.042 km and 171.760 km, respectively). Tan et al. [18] investigated the differences in training characteristics of finishers and non-finishers in a 161 km race. They reported that finishers tended to have longer training sessions compared to non-finishers, even though the weekly running distances were similar. This suggests the importance of longer training sessions for ultramarathon preparation. As stated previously, the runner had participated in several ultramarathons, including 24 h ultramarathons, in the past but had started training about half a year ago. The longest training session before the race was less than half of the aim (200 km). We speculated that the running speed reduction during the latter half of the race may have been because of insufficient training. In other words, the longest training session before the race was too short relative to the aim.

There are several limitations. First, the findings of the present case study may not be generalizable as it included only one male recreational ultramarathon runner. Higher CHO intake would be required for elite and international level 24 h ultramarathon races. Second, we did not evaluate the changes in body weight, body composition, and muscle glycogen content before and after the race. In addition, we did not measure CHO intake, gastrointestinal function (absorption capacity), and integrity (epithelial injury) during the training period. Therefore, a comprehensive analysis of the nutritional status and gastrointestinal status could not be performed. Third, because ultraendurance sports, such as 24 h ultramarathon races, cause cardiac autonomic adjustments [19] and late cardiovascular drift [20], there is a limitation that we used heart rate to estimate physiological load during the 24 h. However, measuring portable oxygen uptake in a real race is impractical.

## 5. Conclusions

The runner had adequate CHO consumption before and during the race and maintained his blood glucose levels throughout the 24 h. However, his running speed decreased during the middle and late stages of the race. The relative intensity during the first half of the race was low, suggesting that the running speed reduction was not due to the pacing strategy. The longest training session before the race was too short relative to the aim (80 km vs. 200 km).

For optimal 24 h ultramarathon performance, the “big three” strategies of training, nutrition, and pacing are all important. However, the performance level estimated based on previous studies may be achievable even with insufficient training, as long as the nutritional and pacing strategies are appropriate.

## Figures and Tables

**Table 1:** Nutritional status before the 24 h ultramarathon.

	Baseline Diet	Pre-Race Diet	Pre-Race Meal
Energy (kcal)	2357 ± 131	2759 ± 223	1224
Protein (g)	123 ± 23	91 ± 9	26
Protein (g/kg)	1.8 ± 0.3	1.3 ± 0.1	0.4
Protein (%energy)	21 ± 4	13 ± 2	8
Fat (g)	65 ± 13	49 ± 17	12
Fat (g/kg)	1.0 ± 0.2	0.7 ± 0.2	0.2
Fat (%energy)	25 ± 5	16 ± 5	9
CHO (g)	322 ± 62	492 ± 57	252
CHO (g/kg)	4.7 ± 0.9	7.2 ± 0.8	3.7
CHO (%energy)	54 ± 8	71 ± 4	83

Data of baseline and prerace diet are presented as means and standard deviations. CHO, carbohydrate.

**Table 2:** Nutritional status during the 24 h ultramarathon.

	0–6 h	6–12 h	12–18 h	18–24 h	Total (0–24 h)
Energy (kcal)	1778	1702	1324	1518	6322
Energy (kcal/h)	296	284	221	253	263
Protein (g)	40	36	33	41	151
Protein (g/kg)	0.6	0.5	0.5	0.6	2.2
Protein (g/h)	6.7	6.1	5.5	6.9	6.3
Fat (g)	23	22	33	35	113
Fat (g/kg)	0.3	0.3	0.5	0.5	1.6
Fat (g/h)	3.9	3.7	5.5	5.8	4.7
CHO (g)	366	342	216	250	1174
CHO (g/kg)	5.3	5.0	3.2	3.6	17
CHO (g/h)	61	57	36	42	48
Fluid (mL)	1650	1650	1500	1500	6300
Fluid (mL/h)	275	275	250	250	263

CHO, carbohydrate.

## Data Availability

The data presented in this study are available on request from the corresponding author.
